# Predominance of sperm motion in corners

**DOI:** 10.1038/srep26669

**Published:** 2016-05-23

**Authors:** Reza Nosrati, Percival J. Graham, Qiaozhi Liu, David Sinton

**Affiliations:** 1 Department of Mechanical and Industrial Engineering, University of Toronto, Toronto, ON, M5S 3G8, Canada

## Abstract

Sperm migration through the female tract is crucial to fertilization, but the role of the complex and confined structure of the fallopian tube in sperm guidance remains unknown. Here, by confocal imaging microchannels head-on, we distinguish corner- vs. wall- vs. bulk-swimming bull sperm in confined geometries. Corner-swimming dominates with local areal concentrations as high as 200-fold that of the bulk. The relative degree of corner-swimming is strongest in small channels, decreases with increasing channel size, and plateaus for channels above 200 μm. Corner-swimming remains predominant across the physiologically-relevant range of viscosity and pH. Together, boundary-following sperm account for over 95% of the sperm distribution in small rectangular channels, which is similar to the percentage of wall swimmers in circular channels of similar size. We also demonstrate that wall-swimming sperm travel closer to walls in smaller channels (~100 μm), where the opposite wall is within the hydrodynamic interaction length-scale. The corner accumulation effect is more than the superposition of the influence of two walls, and over 5-fold stronger than that of a single wall. These findings suggest that folds and corners are dominant in sperm migration in the narrow (sub-mm) lumen of the fallopian tube and microchannel-based sperm selection devices.

Sperm motion near surfaces plays a key role in natural fertilization, but the role of the complex and three-dimensional (3D) structure of the female tract on sperm migration near surfaces is largely unknown. During the journey to the egg, sperm encounter various rheological, biochemical, thermal and geometrical conditions. Specifically, viscosities range from 1 to over 100 mPa s^1^,^2^, pH ranges from 6.5 to 8.5^1^,^3^, temperatures range from 35 to 38 °C^4^ and confinement on the order of 10 to 100 μm is common^5^,^6^. These variations enable sperm guidance and possible selection mechanisms, namely rheotaxis^7^,^8^,^9^,^10^, chemotaxis^4^,^11^, thermotaxis^12^,^13^, and boundary-following navigation^14^,^15^. With respect to the geometry, the fallopian tube becomes remarkably folded and confined with narrow lumen and corners as the sperm progresses along its journey^5^,^6^. Emerging assisted reproduction methods also employ microconfined geometries to mimic the female tract for *in vitro* sperm selection^16^,^17^,^18^,^19^ and to coordinate fertilization^20^,^21^,^22^,^23^. There is a lack, however, of quantitative understanding of how geometrical complexity and confinement influence sperm motion.

The study of sperm-surface interaction began in 1963, with Rothschild’s discovery of surface accumulation behaviour in bull sperm^24^. This phenomenon has been studied for variety of microswimmers, by considering the effect of geometrical constraints^25^,^26^, hydrodynamic effects^27^,^28^, and flagellar beat pattern^29^,^30^. These studies established that microswimmers, including sperm, accumulate near boundaries mainly due to a combination of hydrodynamic forces^27^,^31^ and steric repulsion^15^. Surface confinement has also been shown to alter the swimming pattern and flagellar waveform of sperm^32^,^33^,^34^. Recently we discovered an intermittent, fully two-dimensional slither swimming mode whereby the full sperm length (~70 μm) oscillates within 1 μm of the surface^32^. Denissenko *et al*.^14^ demonstrated that the migration ability of human sperm in a microchannels depends critically on the channel geometry, with corners contributing to boundary-following navigation. However, previous studies were limited to orthogonal imaging of the channel with a large depth of field (imaging a 1D distribution across the channel width), thus, could neither resolve nor quantify the distribution of sperm swimming close to a wall vs. those swimming close to a corner (requiring a 2D distribution across the channel).

Here, we resolve and quantify corner vs. wall vs. bulk swimmers by confocal imaging microchannels head-on. Our results demonstrate the strong preference of the bull sperm (~75 μm in length) to swim near corners (within 15 μm of the intersection of two walls) in rectangular microchannels, with local areal concentrations as high as 200-fold that of the bulk. The relative degree of corner-swimming preference is heavily dependent on channel size and shape, with channels above 200 μm resulting in similar corner, wall and bulk distributions. Furthermore, we characterize the effect of viscosity and pH on corner-swimming preference of sperm in square microchannels. Together, combined corner and wall swimmers account for over 95% of the sperm distribution in small rectangular channels, which is similar to the percentage of wall swimmers in similarly-sized circular microchannels. In the context of reproduction, this strong corner-swimming behavior highlights the role of geometrically complex and confined environment within the female tract on sperm navigation. In the context of assisted reproduction, corner-directed sperm motion plays a dominant role in sperm migration within microchannel-based sperm selection devices.

## Results and Discussion

### Corner-swimming in square microchannels

**Figure 1:**
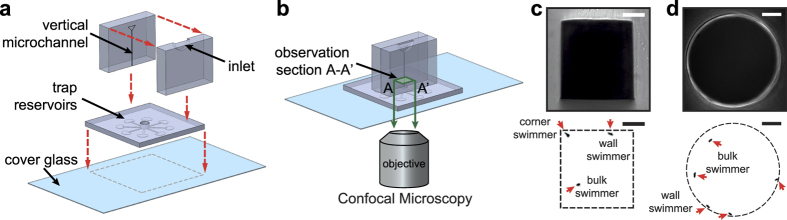
Microfluidic device for quantifying cross-sectional distribution of sperm in microchannels. (**a**) Schematic view of the device: a microchannel is vertically aligned with an observation chamber in the horizontal layer. (**b**) A unique head-on microchannel confocal microscopy approach was used for imaging. The shallow focal plane was focused at the entry of the vertical microchannel to the observation chamber and then moved 20 μm inside the channel. A representative bright-field image of the microchannel cross-section (top) and fluorescence image of sperm in the microchannel cross-section (bottom) for (**c**) rectangular and (**d**) circular microchannels. White dash lines in fluorescence images indicate the microchannel wall, acquired using bright-field images, and red arrows point to sperm. Scale bars represent 30 μm and 45 μm for rectangular and circular microchannels, respectively.

The distinction and quantification of the swimming preference of sperm in the microchannel cross-sectional area was enabled by head-on confocal imaging, as shown in Fig. 1. The device comprises of a vertical and a horizontal layer (see Methods). The vertical layer, a 7.2 mm long microchannel, was aligned with the horizontal layer such that the microchannel cross-section was located at the center of a cylindrical chamber in the horizontal layer (Fig. 1a). The device was pre-filled with a biologically relevant buffer, mimicking the natural environment *in vivo*^35^. Flow was inhibited by the dead-end structure of the device as well as relatively high fluid viscosities. The vertical channel exits into the cylindrical chamber which leads to eight horizontal trap reservoirs. The trap reservoirs use ratchet shape geometries^15^,^36^,^37^ to prevent sperm from re-entering the chamber. To characterize the effects of geometry and confinement, both rectangular and circular cross-section vertical channels, of varying dimensions, were tested. This characterization is relevant to both (*i*) sperm migration through the folded epithelium of the fallopian tube and the effect of confinement on sperm guidance *in vivo*, as well as (*ii*) sperm migration through microfluidic devices that generally have rectangular or square cross-sections.

Confocal microscopy was used to image migrating sperm through the vertical channel, at a cross-section 20 μm above the channel exit to the cylindrical chamber (Fig. 1b). The thickness of the focal plane was confined to 12.8–14.0 μm along the axial channel direction, to image a representative vertical channel cross-section, well before the channel exit. Sequences of bright-field and green fluorescence images with 500 ms interval were recorded for 30 min (see Movie S1 and S2). Bright-field (Fig. 1c,d top) and fluorescence (Fig. 1c,d bottom) imaging were used to locate channel walls and sperm, respectively. In contrast to previous imaging layouts that only give a 1D distribution by using the side-view of the microchannel, the method presented here images the channel head-on, providing the full 2D distribution of sperm. As a result we can accurately distinguish (*i*) a bulk swimmer at the center of the channel from a wall swimmer at the central part of the wall and (*ii*) a wall-swimming sperm at the center of the wall from a corner-swimming sperm at the channel corner.

**Figure 2:**
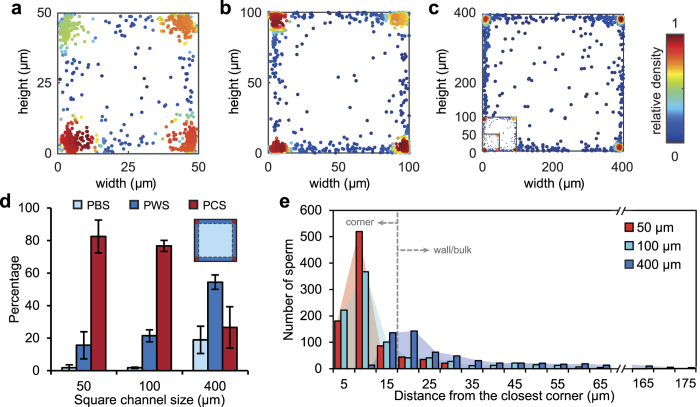
Corner-swimming preference of sperm in square microchannels. Cross-sectional distribution of 1176, 1457, and 1188 bull sperm in square microchannels with side-lengths of (**a**) 50 μm, (**b**) 100 μm, and (**c**) 400 μm, respectively. For comparison, the 50 μm and 100 μm channels are shown to scale inset in the bottom left corner of the 400 μm channel plot. The color bar represents the relative density of sperm in each graph. (**d**) Percentage of bulk swimmer (PBS), percentage of wall swimmer (PWS), and percentage of corner swimmer (PCS) sperm as a function of microchannel size. Wall-swimming sperm values do not include corner swimmers. Each point represents experiments with at least three samples with a minimum of 874 sperm imaged in each experiment. Values are reported as mean ± s.d. (**e**) Histograms of sperm distance from the closest corner for each case. A total number of 800 sperm is included in each case.

Figure 2 shows the cross-sectional distribution of sperm in square microchannels with side-lengths of 50 μm, 100 μm, and 400 μm. Sperm within 15 μm of only one wall were considered as wall swimmers and sperm within 15 μm of two walls were considered as corner swimmers, all other sperm were considered as bulk swimmers. The 15-μm threshold for wall proximity is based on previous works, indicating that microswimmers, including sperm, are most densely accumulated within 15–20 μm of the surface with their concentration decaying exponentially with distance from the surface^38^,^39^,^40^,^41^. The 2D scatter distributions of sperm across the microchannels are shown in Fig. 2a–c with the relative density of sperm indicated via the color bar (for each plot). The plots of Fig. 2a,b are shown inset in Fig. 2c to clarify the geometries. The results indicate the predominance of corner-swimming as points with red and green colors in the plots indicate that many sperm swim near the corners during the imaging period.

Figure 2d quantifies the percentage of bulk-swimming (PBS), percentage of wall-swimming (PWS), and percentage of corner-swimming (PCS) sperm as a function of the microchannel size. The corner-swimming tendency was predominant in both the 50- and 100-μm channels, accounting for 82% and 76% of sperm, respectively. In the significantly larger 400-μm channel, corner-swimming accounting for 27% of sperm. In contrast to corner-swimming, wall-swimming increased with channel size, specifically, with 50, 100 and 400 μm microchannels having 16%, 22% and 54% wall-swimming sperm, respectively. Similarly, bulk-swimming also increased from less than 2% for 50 μm and 100 μm channels to 19% in 400 μm channels. Thus, as the channel size increases, the concentration of corner swimming sperm decreases, while the concentration of wall and - to a lesser extent - bulk swimming sperm increases. This shift is mainly due to larger channel perimeter and higher area-to-perimeter ratio.

In terms of areal concentration, the relative area corresponding to corner-swimming regions decreases as channel size increases (36%, 9% and <1% for 50, 100, and 400 μm channels respectively). Although only 27% of sperm swim in the corners of a 400 μm channel, the corner regions correspond to less than 1% of the channel cross-sectional area. Local areal concentrations in the corners reach over 200-fold that of the bulk for both 100 and 400 μm channels (227- and 213-fold, respectively).

Figure 2e shows histograms of sperm distance from the closest corner, indicating a sharp right-skewed distribution for all channel sizes. For channels with 50 μm and 100 μm size, the maximum number of sperm was captured within 10–15 μm of the closest corner. For channels of 400 μm in size, the distribution had a long tail and a broad peak shifted to 20–25 μm from the closest corner. Furthermore, Fig. 2e shows that wall-swimmers are not uniformly distributed along the walls. Rather, the wall swimmers are more densely concentrated near the corner regions, with frequency decreasing along the wall.

Both wall- and corner-swimming preferences originate from hydrodynamic interaction of sperm with surfaces^41^,^42^. It is clear from the experiments that the corner effect is stronger than simply the superposition of the influence of two walls. A simple superposition would predict a two-fold increase in relative density, whereas results here demonstrate over 5-fold increase in corners (maximum relative density of 0.51) relative to walls (maximum relative density of 0.12), as seen in Fig. 2a. Also from the perspective of flow theory, near-wall swimming hydrodynamics are well approximated by a single image of a dipole^39^,^43^ and subsequently an attractive force toward the surface^38^,^41^,^42^. The presence of a corner, however, requires a second reflection of both the sperm flow field and the image^44^,^45^. The resulting hydrodynamic effect is nonlinear, and greater than a direct combination of two walls. Recent computational modelling indicated that a simple bacterium near two orthogonal walls oscillates along one of the walls while remaining in close proximity to the corner, demonstrating that a corner has an effect distinct from the superposition of two walls^46^. These findings suggest that multiple physical boundaries confine the 3D swimming trajectories of sperm to 1D trajectories along the corners, amplifying the progressive motion.

### Corner-swimming in rectangular microchannels

**Figure 3:**
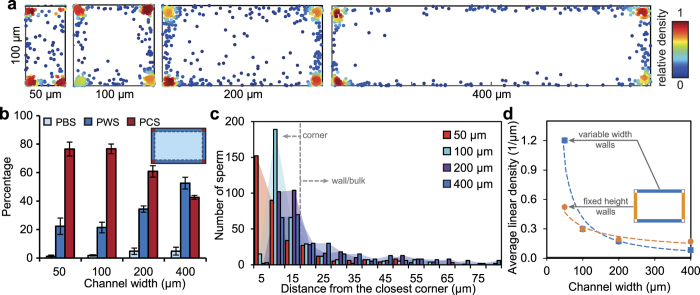
Corner-swimming preference of sperm in rectangular microchannels with varying width, all 100 μm in height. (**a**) Cross-sectional distribution of 929, 1068, 1023, and 956 bull sperm in rectangular microchannels of 100 μm in height and 50 μm, 100 μm, 200 μm, and 400 μm in width. The color bar represents the relative density of sperm in each graph. (**b**) PBS, PWS and PCS sperm as a function of microchannel width. Wall-swimming sperm do not include corner-swimming sperm. Each bar represents at least three experiments with a minimum of 589 sperm imaged in each. Values are reported as mean ± s.d. (**c**) Histograms of sperm distance from the closest corner for all rectangular microchannels. A total number of 500 sperm is included in each case. (**d**) Average linear density - percentage of wall swimmers per unit length of wall – plotted for both the fixed height walls and the variable width walls, for all four cases.

To further analyze the corner-swimming preference, 100-μm high rectangular microchannels with widths of 50, 100, 200, and 400 μm were tested. The 2D scatter distributions of sperm, with red points concentrated at the corners, indicate the strong corner-swimming preference (Fig. 3a). Similar to square microchannels, the corner-swimming preference decreased with microchannel size (Fig. 3b). Both 100 × 50 μm and 100 × 100 μm devices demonstrated a strong corner-swimming preference, with 76% corner-swimming and 22% wall-swimming sperm. By increasing only the channel width to 200 and 400 μm, corner-swimming decreased to 60% and 42%, respectively. In contrast to corner-swimming, wall-swimming increased with channel width to 34% in 200-μm wide channels and even higher to 52% (24% higher than the corresponding corner-swimming) in 400-μm wide channels. Thus, as the channel width increases with height fixed, the concentration of wall swimmers increases at the expense of corner swimmers. A shift into the bulk is detectable, but not as significant as in square channels since rectangular channel cross-sections have relatively more boundary (lower area-to-perimeter ratio).

In terms of areal concentration, the relative area corresponding to corner-swimming regions decreases as channel width increases (18%, 9%, 4.5%, and 2.25% for100-μm high microchannels with widths of 50, 100, 200, and 400 μm, respectively). Similar to square channels, the results indicate a strong corner-swimming preference for larger channels. Specifically, local areal concentrations in the corners reach over 150-fold that of the bulk for 100, 200 and 400 μm channels (227-, 169-, and 255-fold, respectively).

Histograms of sperm distance from the closest corner (Fig. 3c) show a similar trend to that of square channels (Fig. 2c), with wall swimmers most densely concentrated near the corner regions. For 50 and 100 μm wide channels, the maximum number of sperm was captured within 5 and 10 μm of the closest corner, respectively. The peak was broadened and shifted up to 15 μm for both 200 and 400 μm widths. For rectangular channels, the higher concentration of wall swimmers near the corners results in differing densities of sperm on short and long channel walls. Figure 3d plots the average linear density of wall-swimming sperm (percentage of wall-swimming per unit length of the microchannel wall) for both fixed height and variable width walls. Average linear density decreased by 42% and 75% along the fixed height and variable width walls, respectively, as the channel width increases from 50 to 100 μm then plateaus for larger sizes. The decreasing trends of average linear density with channel size indicates that the increase in wall-swimming preference for larger channels is mainly due to larger channel perimeter, and not due to capturing higher number of sperm per unit length of the wall. Higher density of wall-swimming sperm on short walls is attributed to the relative proximity to the corners, where sperm are densely concentrated (Fig. 3c). Additionally, the results indicate that the wall-swimming preference for sperm more than 100 μm from the wall (about one body length) is negligible as the linear density plateaus for channels wider than 200 μm.

The corner- and wall-swimming preferences reported here are not attributed to either hyperactivation or changes in swimming velocity of boundary-following sperm. First, hyperactivation could not have induced corner or wall swimming, as only progressively motile sperm will be able to swim a relatively long distance (7.2 mm, 100 body lengths) to reach the imaging section. Second, the imaging period (30 min) is long enough to allow slow swimmers to reach the imaging section.

### Swimming preference in circular microchannels

**Figure 4:**
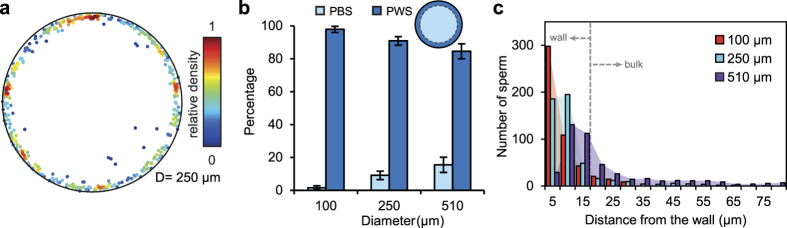
Swimming preference of sperm in circular microchannels. (**a**) A representative cross-sectional distribution of 596 bull sperm in circular microchannels of 250 μm in diameter. Color bar represents the relative density of sperm in each graph. (**b**) PBS and PWS sperm as a function of microchannel diameter. Each points represents experiments with at least three samples with a minimum of 494 sperm imaged in each of the experiments. Values are reported as mean ± s.d. (**c**) Histograms of sperm distance from the wall for all circular microchannels. A total number of 494 sperm is included in each case.

Figure 4 shows the swimming preference of sperm in circular microchannels with 100-, 250- and 510-μm diameter. Similar to rectangular microchannels, sperm exhibited a strong wall-swimming preference in circular microchannels (Fig. 4a). The wall-swimming preference decreased as the diameter of the microchannel increased, as shown in Fig. 4b. Specifically, wall-swimming linearly decreased from 98% to 91% and 85% by increasing the channel diameter from 100 to 250 and 510 μm, respectively. In terms of wall-swimming tendency and channel diameter, this is a strong linear trend (R^2^ = 0.99), reflecting a straightforward dependence on area-to-perimeter ratio in the absence of corners. Importantly, the percentage of wall-swimmers for circular microchannels is comparable with combined percentage of wall and corner swimmers in square and rectangular microchannels with similar hydraulic diameters.

The proximity of imaged sperm to the circular microchannel wall is plotted in Fig. 4c. Similar to the distribution obtained for square and rectangular channels, the maximum number of sperm was captured within 5 μm of the wall for the smallest channels, with the peak shifting to 10–15 μm for larger channels. The preferred range of 10–20 μm is well established as the equilibrium distance for accumulation at planar surfaces^38^,^39^,^40^,^41^. Why sperm accumulate more closely to the surface of small channels, as observed here, is not fully clear. We expect the tighter surface accumulation is due to the proximity of the opposite wall, specifically, when geometrical confinement is comparable to the hydrodynamic interaction length-scale - both on the order of 100 μm (about one body length). Thus, sperm travel closer to the walls in smaller channels, where the confinement matches that of the hydrodynamic length-scale.

### Influence of media on corner-swimming

**Figure 5:**
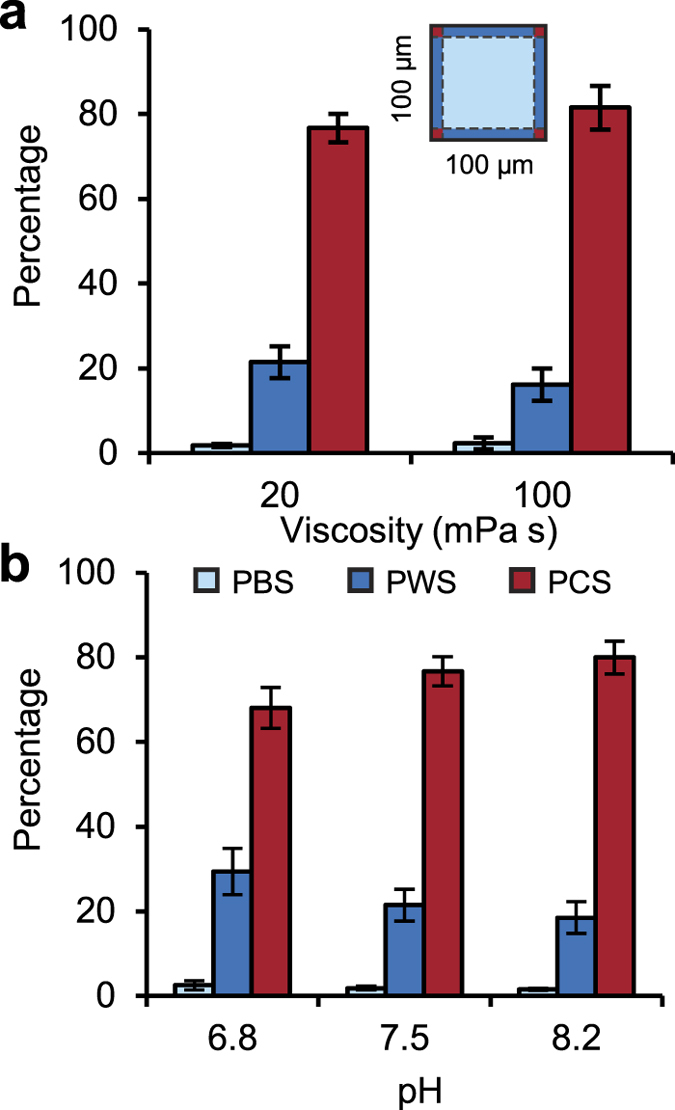
Effects of the swimming medium on corner-swimming preference of sperm in 100 × 100 μm microchannels. PBS, PWS, and PCS sperm as a function of (**a**) viscosity and (**b**) pH of swimming media. Wall-swimming sperm do not include corner-swimming sperm. Each bar represents at least three experiments with a minimum of 874 sperm imaged in each. Values are reported as mean ± s.d.

To study the influence of media properties on corner-swimming preference, we tested 100 × 100 μm devices filled with buffers with viscosities of 20 and 100 mPa s (with pH of 7.5) and buffers with pH of 6.8, 7.5, and 8.2 (with a viscosity of 20 mPa s). Corner-swimming increased with both viscosity and pH, as shown in Fig. 5. Specifically, corner-swimming slightly increased from 76% to 82% by increasing the viscosity from 20 to 100 mPa s, with slight decrease in wall-swimming from 21% to 16%, respectively. Similarly, corner-swimming slightly increased from 68% to 76% and 79% by increasing the pH from 6.8 to 7.5 and 8.2, respectively (with corresponding wall-swimming of 29%, 21%, and 18%). The increase in corner-swimming preference by increasing viscosity and pH of the swimming medium is attributed to the change in flagellar waveform and beating pattern which alters the hydrodynamics of sperm motion^2^,^32^,^47^. Specifically, increased viscosity increases the drag forces that act on the sperm and as a result suppresses the torsion and yaw in the swimming trajectory, while increased pH alters the flagellar waveform and increases sperm motility. Both viscosity and pH increase surface accumulation and potentially corner-swimming. The results indicate the corner-swimming remains predominant across the physiologically relevant range of the viscosity^1^,^2^ and pH^1^,^3^ *in vivo*.

## Conclusion

We resolved and quantified corner- vs. wall- vs. bulk-swimming bull sperm in both rectangular and circular channels by confocal imaging microchannels head-on. Our results demonstrate the strong preference of sperm (~75 μm in length) to swim near corners (within 15 μm of the intersection of two walls) in rectangular microchannels. The remarkably folded and confined lumen of the fallopian tube narrows towards the egg and presents a swimming environment far more intricate than a single flat surface. The predominance of corner-swimming highlights the role of this increasing complexity of the fallopian tube in sperm guidance.

The corner-swimming preference originates from hydrodynamic interactions between sperm and surfaces. We demonstrate that the corner accumulation is more than the superposition of the influence of two walls, and over 5-fold stronger than that of a single wall. As channel size increases, the concentration of corner swimming sperm decreases, while the concentrations of wall swimming and - to a lesser extent - bulk swimming sperm increase. This shift in corner-swimming is mainly due to larger channel perimeter and higher area-to-perimeter ratio. In terms of area, local areal concentrations in the corners reach over 200-fold that of the bulk for both 100 and 400 μm square channels. The distribution of the wall-swimming sperm along the wall is non-uniform with wall-swimmers most densely concentrated near corner regions. Testing with different media properties indicated that corner-swimming remains predominant across the physiologically relevant range of the viscosity and pH *in vivo*. These findings suggest that multiple physical boundaries confine the 3D swimming trajectories of sperm to 1D trajectories along corners and folds, amplifying the progressive motion.

The results demonstrate that boundaries play a significant role in sperm guidance, as over 95% of the sperm traverse the channel near the walls and corners in small channels. The percentage of wall-swimmers for circular microchannels is comparable with combined percentage of wall and corner swimmers in square and rectangular microchannels with similar hydraulic diameters. For all three channel shapes studied here, we observed a tighter surface accumulation behavior when geometrical confinement is comparable to the hydrodynamic interaction length-scale - both on the order of 100 μm (about one body length). Thus sperm travel closer to the walls in smaller channels, where the confinement matches that of the hydrodynamic length-scale.

In the context of natural reproduction, these findings highlight the role of the geometrical complexity and confinement, typical of the fallopian tube in the isthmus and the ampulla, on sperm navigation. The highly folded epithelium of the tract results in narrow lumen and corners which are likely to confine the 3D swimming trajectories of sperm to 1D trajectories along the corners, amplifying the progressive motion – potentially providing a route to the egg. In addition, the changes in chemical and rheological properties of the fallopian tube can also serve as a mechanism to influence the swimming preference of sperm with respect to the boundaries and direct the sperm towards the oocyte. In the context of assisted reproduction, corners are common in emerging microchannel-based sperm selection methods. The predominance of corner swimming potentially inspires improved selection strategies for *in vitro* fertilization. These findings highlight the dominant role of corner-directed sperm migration within microchannel-based sperm selection devices. The corner-swimming preference is of profound importance in developing new microfluidic technologies for motility-based sperm selection, since rectangular microchannels are the most established geometries to fabricate a microfluidic device.

## Methods

### Device fabrication

A microfluidic device was designed and fabricated to quantify the distribution of sperm in the cross-sectional area of a microchannel, as shown in Fig. 1. The device consisted of a vertical and a horizontal layer. The vertical layer contained an inlet and a microchannel. The semi-circular inlet served as a guide for sperm to swim into the microchannel. The vertical layer was aligned with the horizontal layer such that the microchannel cross-section was located at the center of the observation. The vertical channel exits into a cylindrical chamber (1.5 mm in diameter) in the horizontal layer. This chamber leads to eight trap reservoirs which use ratchet shape geometries^15^,^36^,^37^ to prevent sperm from re-entering the chamber. The ratchets are arrowhead–shaped with a concave sections around their entrance to redirect sperm back in to the trap, ensuring a unidirectional flux of the cells into the traps. The microfluidic device was designed in AutoCAD and printed on a photomask (CAD/Art Services, Inc., OR, USA). For devices with rectangular microchannels, masters with 50 μm, 100 μm, 200 μm, and 400 μm heights were fabricated from negative SU-8 photoresists (MicroChem, Newton, MA, USA) using standard soft lithography technique^48^. The master for the bottom layer was always fabricated using SU-8 2075 with features 100 μm in height. Both layers were fabricated using Poly-dimethylsiloxane (PDMS) (Silgards 184: Dow Corning, MI, USA) substrate with 1:10 mixing ratio. A 1.5 mm Miltex Dermal Biopsy punch was used to punch a hole at the center of the horizontal layer. The geometry of the respective top and bottom layers were closed by bonding a plain layer of PDMS and a Micro Cover Glass No. 1 (Rectangular, 22 × 50 mm, VWR, PA, USA) using a hand-held corona treater (BD-20AC, Electro-Technic Products Inc., IL, USA). The microchannel in the vertical layer was cut to be 7.2 mm in length (including the inlet part), manually aligned at the center of the hole in the horizontal layer, and bonded using uncured PDMS.

For devices with circular microchannels, the inlet was designed as a disk 3 mm in diameter and 0.8 mm thick. The master for the inlet layer was fabricated by cutting a plastic disk from a 0.8 mm thick sheet of Polymethyl methacrylate (PMMA) (Plastic Word, Toronto, Canada) using a M-360 CO_2_ laser, the disk was then bonded to a petri dish using Chloroform (Sigma-Aldrich Corp, MO, USA). Sufficient PDMS was poured to form an 8 mm thick layer, then a Miltex Dermal Biopsy punch was used to punch a 1.5 mm diameter hole at the center of the inlet. Tubes 7.2 mm in length and inside diameter of 100 μm, 250 μm, and 510 μm (1.58 mm in outside diameter, biocompatible, DuPont FEP Tubing, Fisher Scientific, Canada) were pushed through the punched hole to form circular microchannels. The bottom layer was fabricated and the layers were bonded similarly as described for devices with rectangular microchannels.

### Buffer preparation

HEPES-buffered saline (HBS) (135 mM NaCl, 5 mM KCl, 12 mM D-Glucose, 25 mM HEPES, 0.75 mM Na_2_HPO_4_·2H_2_O) supplemented with 1 mg/mL Poly(vinyl Alcohol) (PVA) with 0.5% and 0.875% Methyl cellulose (MC) (M0512; Sigma-Aldrich Corp, MO) was used to prepare non-Newtonian viscoelastic buffer with nominal viscosity of 20 and 100 mPa s at 20 °C according to manufacturer’s manual, respectively. The viscosity of the buffer with 0.5% and 0.875% MC at 37 °C were measured using a Brookfield LVDV-E digital viscometer (Brookfield Engineering Laboratories, Inc., MA, USA) with spindle LV2 at 100 r.p.m. to be 18.95 ± 0.15 and 88.53 ± 1.40 mPa s, respectively. All viscosities values stated in the text are nominal values at 20 °C unless otherwise mentioned. Finally, a 1 M solution of NaOH (VWR, PA, USA) was used to adjust the buffer pH to 6.8, 7.5, and 8.2. The buffer was stored at 4 °C and used within two weeks of preparation. Buffer with 0.5% MC and pH of 7.5 was used for all of the experiments unless otherwise stated.

### Semen sample preparation

Bull semen with approximate concentration of 50 million sperm per milliliter and 50% motility were purchased in 500 μL straws (ABS Global Inc., Canada) and stored in liquid nitrogen. Before the experiments, bull semen was thawed in a 37 °C water bath for 5 min and extracted using an artificial insemination syringe. To stain live sperm with green fluorescence, 10 μL of 50-fold diluted solution of SYBR14 (Component A, LIVE/DEAD sperm viability kit, L-7011; Invitrogen, NY, USA) was added to 500 μL of semen and incubated at 37 °C for 10 min. This staining step was required to ensure that we can leverage the relatively thin focal plane in confocal microscopy to capture sperm while they swim normal to the focal plane. The bull semen was kept at 37 °C at all times, and experiments were conducted within 10 min of staining.

### Experimental procedure

The device was filled by submerging it in buffer and applying vacuum pressure (−30 psi) for at least 1 hour and stored for about 1 hour inside a 37 °C incubator until use. All experiments were then performed at room temperature. Previous works^49^,^50^,^51^,^52^ have demonstrated that sperm motility characteristics at room temperature remain comparable to sperm motility at 37 °C, for up to 3 hours. The chip was mounted to a Nikon A1 confocal microscope stage. A 40× magnification microscope objective (NA = 0.6, WD = 3.6 mm) was used for all of the confocal microscopy experiments except for the ones conducted with devices with the microchannel dimension of 400 μm or larger where a 10× magnification microscope objective (NA = 0.5, WD = 4.0 mm) was used. A 1.2 AU pinhole was used during the confocal microscopy experiments, resulting in 12.8 μm and 14.0 μm depth of the focal plane for 40× and 10× magnification objectives, respectively. The focal plane was positioned along the vertical microchannel, 20 μm above the channel exit to the cylindrical chamber, ensuring only sperm inside the vertical channel cross-section were being imaged (section A-A′ in Fig. 1b).

Following this step, 30 μL of fluorescently labelled semen sample was pipetted into the inlet of the device. Since the semen sample was introduced at the entry of a prefilled dead-end microchannel, no flow was maintained within the microchannel during the experiments and sperm swam along the channel based on their own preference (tested using a prefilled device with buffer containing fluorescent particles). Progressively motile sperm must swim 7.2 mm along the vertical microchannel to reach the imaging section. After observing the first sperm in the microchannel cross-section in live mode of the microscope software, confocal imaging system was used to capture sequences of bright-field and green fluorescence images with 500 ms intervals for 30 min. The bright-field images was used to recognize the channel walls in the fluorescence images. The freely available image processing software ImageJ was used to manually locate sperm across the microchannel and a custom written script in Matlab was used to process the data. It is noteworthy that the sperm swim aligned with the wall and the center of the sperm head was tracked for each point. Sperm with distance smaller than 15 μm from a wall were considered as wall swimmer sperm and sperm with distance smaller than 15 μm from two walls were considered as corner swimmer sperm, all other sperm were considered as bulk swimmer sperm. The ratio of corner swimmer, wall swimmer, and bulk swimmer sperm to total number of imaged sperm was stated as percentage of corner swimmers (PCS), percentage of wall swimmers (PWS), and percentage of bulk swimmers (PBS), respectively.

## Additional Information

**How to cite this article**: Nosrati, R. *et al*. Predominance of sperm motion in corners. *Sci. Rep.* **6**, 26669; doi: 10.1038/srep26669 (2016).

## Supplementary Material

Supplementary Information

Supplementary Movie 1

Supplementary Movie 2
