# Supplementary material for: Predominance of sperm motion in corners
Source: Sci Rep. 2016 May 23;6:26669. doi: 10.1038/srep26669 (PMC4876399; doi:10.1038/srep26669)
Supplement: Supplementary Information [file srep26669-s1.pdf]

Supplementary Information file

Predominance of sperm motion in corners

Reza Nosrati[1], Percival J. Graham [1], Qiaozhi Liu [1], and David Sinton[*,1]

[1] Department of Mechanical and Industrial Engineering, University of Toronto, Toronto, ON, M5S 3G8, Canada.

[*] Corresponding author:
Prof. David Sinton
Department of Mechanical and Industrial Engineering, University of Toronto, 5 King's College Rd., Toronto, ON, Canada, M5S 3G8.
Phone: +1 (416) 978-1623.
E-mail: sinton@mie.utoronto.ca

Movie S1. Corner-swimming of sperm in 100 µm square microchannels. The scale bar represents 30 µm and the video speed is 5x real-time.

Movie S2. Wall-swimming of sperm in 250 µm circular microchannels. The scale bar represents 45 µm and the video speed is 5x real-time.
